# Comparison between Submucosal Tunneling Endoscopic Resection and Endoscopic Submucosal Dissection for Prepyloric Submucosal Tumors: A Case-Matched Controlled Study

**DOI:** 10.1155/2023/5931360

**Published:** 2023-03-01

**Authors:** Wengang Zhang, Jiafeng Wang, Ningli Chai, Enqiang Linghu

**Affiliations:** Department of Gastroenterology, The First Medical Center of Chinese PLA General Hospital, Beijing 100853, China

## Abstract

**Objectives:**

Endoscopic submucosal dissection (ESD) has become a well-established treatment method for gastric submucosal tumors (SMTs). However, there existed some challenges to perform ESD for prepyloric SMTs on account of the special location. Recently, submucosal tunneling endoscopic resection (STER) provided a novel option for prepyloric SMTs. This study aimed to make a comprehensive comparison between prepyloric STER (P-STER) and ESD for the treatment of prepyloric SMTs.

**Methods:**

Patients with prepyloric SMTs undergoing P-STER treatment between January 2016 and October 2021 were retrospectively reviewed and individually matched at 1 : 1 ratio with those with ESD treatment according to lesion size, lesion location, pathologic diagnosis, lesion origin, and surgery date, forming P-STER and ESD group, respectively. A sample size of 12 patients was collected for each group. Treatment outcomes including resection time, en bloc resection rate, complete resection rate, and postoperative hospital stay as well as occurrence of complications were evaluated.

**Results:**

Compared with ESD group, P-STER group got shorter resection time (52.50 minutes for ESD group vs. 38.67 minutes for P-STER group, *P* = 0.001), shorter postoperative hospital stay (7.00 day for ESD group vs. 5.50 day for P-STER group, *P* = 0.008), and lower rate of postoperative abdominal pain (50.00% for ESD group vs. 8.33% for P-STER group, *P* = 0.025). No complication was encountered in P-STER group, whereas one patient with postoperative bleeding was found in ESD group.

**Conclusions:**

For the treatment of prepyloric SMTs, P-STER appeared to be a more effective endoscopic technique compared with ESD, although further randomized controlled trials were warranted.

## 1. Introduction

To date, endoscopic submucosal dissection (ESD) is a well-accepted method for removing the gastric submucosal tumors (SMTs) in China [1–3]. However, to a certain extent, it is time-consuming and difficult to perform ESD for prepyloric SMTs given the special location. First, the prepyloric SMTs were often perpendicular to the resection knife under endoscopy, increasing the risk of muscularis propria (MP) damage and operation difficulty. Second, the postoperative wound tended to involve the pylorus ring, which might affect the function of pyloric sphincter. Therefore, in 2017, we first introduced the prepyloric submucosal tunneling endoscopic resection (P-STER) for a case of SMT [4]. P-STER technique has the potential to lower the operation time and difficulty by establishing a submucosal tunnel, which can slow down the diffusion of the submucosal liquid cushion and provide a clear vision for endoscopic resection; moreover, the integrity of mucosa above postoperative wound can decrease the influence of the possible gastric-wall defect (GWD) and pylorus ring involvement. However, the studies confirming the safety, effectiveness, and advantages of P-STER for SMTs are lacking. The aim of this study, therefore, was to report the outcomes from P-STER, and compare the efficiency between P-STER and ESD for treating prepyloric SMTs.

## 2. Materials and Methods

### 2.1. Patients

This retrospective study was carried out under the Institutional Review Board of Chinese PLA General Hospital. Informed consent was obtained for all patients described. Between January 2016 and October 2021, a total of 54 patients undergoing P-STER or ESD for prepyloric SMT in our hospital. Patient-related and procedure-related data were retrieved from a prospectively maintained database. The inclusion criteria were as follows: (1) patients diagnosed as prepyloric SMTs based on postoperative pathology diagnosis; (2) patients ≥18 years old. The exclusion criteria were as follows: (1) patients under clinical follow-up for less than six months or loss of follow-up. Of note, the choice of ESD or P-STER as the treatment of prepyloric SMT was depended on the operation habits of endoscopists.

### 2.2. P-STER and ESD Procedure

Endoscopic ultrasonography (EUS) was routinely performed to determine the lesion origin. Patients were maintained in the left lateral position, and general anesthesia was administered using mechanical ventilation. All procedures were carried out by three experienced endoscopists with experience with over 300 ESD cases and 300 STER cases. ESD procedure was carried out using the following steps: marking–injection–circumferential incision–submucosal dissection. Of note, the post-ESD wound was closed using metal clips if the GWD occurred during the procedure.

**Figure 1:**
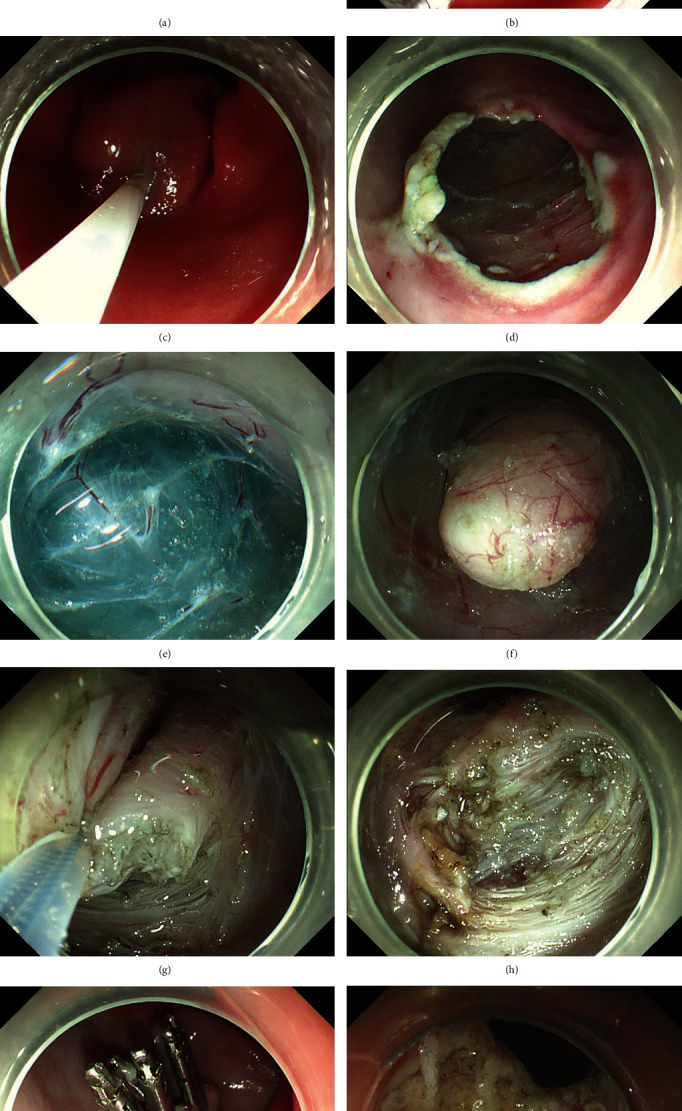
The procedure of P-STER for prepyloric SMTs and the post-ESD wound involved the pylorus ring. (a) The diagram of P-STER for prepyloric SMTs. (b) The appearance of a prepyloric SMT under endoscopy. (c) Submucosal injection at the start site of submucosal tunnel. (d) An inverted T incision was made as the tunnel entrance. (e) Submucosal tunnel was created between the mucosal and muscularis propria (MP) layer. (f) The prepyloric SMT was found in the submucosal tunnel. (g): The basilar part of the prepyloric SMT was resected. (h) The appearance of the postoperative wound. (i) The entry was closed using metal clips. (j) The post-ESD wound involved the pylorus ring.

**Figure 2:**
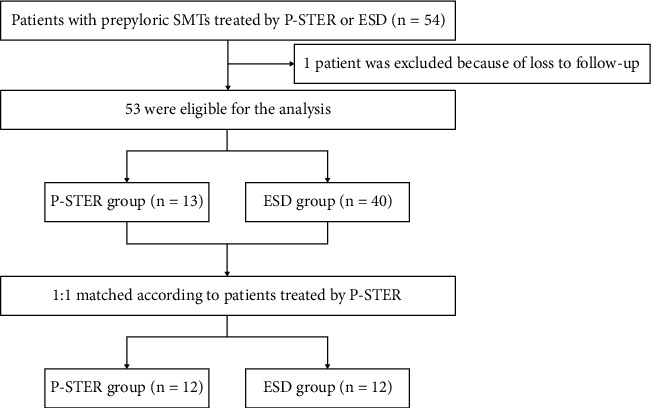
Flowchart of patients enrolled in this study. SMTs: submucosal tumors; P-STER: prepyloric submucosal tunneling endoscopic resection; ESD: endoscopic submucosal dissection.

The P-STER procedure was carried out as follows: (1) Several milliliters of a mixture solution (100 mL saline + 2 mL indigo carmine + 1 mL epinephrine) was injected 3–4 cm proximal to the prepyloric SMTs with an injection needle (NM-4L-1, Olympus; Figures 1(a), 1(b), and 1(c)); (2) an inverted T incision as described previously was made as the tunnel entrance [5] (Figure 1(d)); (3) a tunnel was created between the mucosal and MP layer with the triangular knife and the tunnel ended at 1 cm distal to the prepyloric SMTs (Figures 1(e) and 1(f)); (4) an insulation-tip knife (KD611L, IT2, Olympus), a triangular knife, or a snare (ASM-1-S or ASJ-1-S, Cook, Limerick, Ireland) was used to remove the prepyloric SMT after it was completely exposed (Figures 1(g) and 1(h)); and (5) the incision was closed with clips (HX-610-135, Olympus) after examination of the tunnel (Figure 1(i)). Of note, the endoscopists could chose the full-thickness resection of MP if the lesion originated from the MP layer. The specimen was routinely pinned at a rubber plate for size measurement followed by fixing into formalin for histopathological evaluation.

Moreover, the snare could be used to remove the lesion at the discretion of the endoscopists in both the ESD and P-STER procedures, if operation difficulty was encountered in the final stage of the procedure.

### 2.3. Postoperative Treatments and Follow-Up

Patients in study were observed closely after P-STER or ESD treatment for complications, including perforation, bleeding, and abdominal infection. Corresponding treatment was given once complications were encountered. Patients were kept fasting for 2 days after the procedure, and a liquid diet was followed for an additional 1 day if no complications or postoperative abdominal pain occurred. Diet was gradually restored to normal from the fourth day. Patients would be discharged on the fifth day if there were no complications or postoperative abdominal pain observed, otherwise the hospital discharge would be delayed at the discretion of the endoscopists. Postoperative medications mainly included proton pump inhibitor (PPI) and antibiotics. PPI, including esomeprazole (20 mg, twice a day), rabeprazole (20 mg, once a day), and so on, was required for at least 2 weeks. In terms of antibiotics, levofloxacin, sulperazon, or other third-generation cephalosporin could be administrated. All the 24 patients with prepyloric SMTs involved in this study were arranged with endoscopic follow-up in the local hospital or our hospital in April 2022.

### 2.4. Outcomes and Definitions

Flowchart of the study was shown in detail in Figure 2. In this study, treatment outcomes were analyzed between the P-STER and ESD groups, including en bloc resection, complete resection, GWD, resection time, residue, recurrence, postoperative bleeding, postoperative abdominal pain, postoperative hospital stay, and follow-up time. Resection time was recognized as the time from the submucosal injection in STER procedure or marking in ESD procedure to the withdrawn of endoscopy. Complete resection was defined as the entire removal of lesion in one piece with negative margins. Recurrence meant that the tumor was found again in the original location or location within 1 cm of the original location within the following 6 months postoperatively. Postoperative bleeding was defined as emergence of melena or hematemesis. Postoperative abdominal pain referred to the obvious abdominal pain lasting more than 6 hours, and the pain medications could be prescribed if necessary. Prepyloric SMTs were defined as the SMTs within 3 cm from the pylorus ring.

### 2.5. Statistical Analysis

Comparisons between the two groups were assessed by paired sample *t*-test for continuous variables and the chi-squared for categorical variables. Wilcoxon signed-rank test was used when equal variances were not figured out. Between January 2016 and October 2021, 13 patients with prepyloric SMTs were treated with P-STER in our hospital. Those patients were matched with patients with ESD at a 1:1 ratio according to lesion size (±0.5 cm), lesion location, pathologic diagnosis, lesion origin, and surgery date (±2 years). One out of thirteen patients with prepyloric SMTs undergoing P-STER was excluded because of the failure of lesion location matching with the ESD group (Figure 2). Two-sided *P*-value <0.05 was considered as statistical significance. Parametric data are presented as means. Nonparametric data are expressed as medians.

## 3. Results

### 3.1. Baseline Characteristics of Patients

The baseline characteristics of the 24 patients in this study (12 patients for each group) was depicted in Table 1. There was no significant difference observed in age, gender, lesion size, pathology results, distance from pylorus ring, lesion location, lesion origin, and the appearance under EUS between the two groups studied (*P* > 0.05).

### 3.2. Comparison of Treatment Outcomes between P-STER and ESD Groups

As shown in Table 2, the standard P-STER and ESD treatment outcomes were successfully operated in all the 12 patients, respectively. Among the treatment outcomes, there were no significant difference observed in the usage frequency of the snare during the procedure, GWD, recurrence, postoperative bleeding, and follow-up time (*P* = 0.386, 0.414, 1.000, 0.307, and 0.065, respectively). Only one patient was found with postoperative bleeding in ESD group, and conservative treatment was given. The en-bloc resection was successfully achieved in all the 12 patients in the P-STER and ESD groups respectively. The complete resection appeared to be increased in the P-STER group relative to the ESD group; although, there was no statistical significance [11/12 (91.67%) vs. 9/12 (75.78%), *P* = 0.273]. Of the four patients without complete resection, two were ectopic pancreas, and the other two were gastrointestinal stromal tumors (GISTs) (G1). No recurrence was found during the follow-up for these four patients without complete resection. More importantly, the P-STER group exhibited a significantly less operation time (52.50 minutes for ESD group vs. 38.67 minutes for P-STER group, *P* = 0.001), shorter postoperative hospital stay (7.00 day for ESD group vs. 5.50 day for P-STER group, *P* = 0.008), and lower rate of postoperative abdominal pain (50.00% for ESD group vs. 8.33% for P-STER group, *P* = 0.025), compared with that of the ESD group.

## 4. Discussion

The diagnosis for gastric SMTs are various, including nonneoplastic lesions, benign neoplasms, and potentially and overtly malignant tumors [6]. The nature of the gastric SMTs cannot be determined based on its endoscopic image alone; moreover, a standard endoscopic forceps biopsy typically fails to obtain material adequate for diagnosis [7]. The emergence of ESD made it possible to resect, en bloc, even large mucosal and submucosal lesions [8–10]. In addition, ESD has become a well-established treatment and diagnosis method for gastric SMTs. However, there existed some challenge to conduct ESD for prepyloric SMTs because of the special location. Hence, in 2017, we reported a case of P-STER for a prepyloric SMT.

The present study compared the treatment results of P-STER and ESD for prepyloric SMTs, and showed that patients who received a P-STER underwent a faster operation (Table 2). During the procedures for ESD for prepyloric SMTs, the diffusion of the submucosal liquid cushion was quickly after the circumferential incision, and the approximate vertical angle between the resection knife and MP layer could not be changed. Therefore, ESD for prepyloric SMTs was time-consuming. In comparison, in P-STER procedures, the submucosal injection solutions could be retained in the submucosal layer for a longer time, and the vertical angle between the resection knife and MP layer could be changed by the submucosal liquid cushion; moreover, the submucosal tunnel could provide a clear operation vision for the submucosal dissection. For the foregoing reasons, compared with ESD, P-STER presented a faster operation efficiency. What is more, in ESD procedures, the postoperative wound always involved the pylorus ring (Figure 1(j)), which might affect the function of pyloric sphincter. In contract, in the P-STER procedures, the retained mucosa above the wound might protect the pylorus ring from damage.

On the other hand, the P-STER group exhibited a lower rate of postoperative abdominal pain and shorter postoperative hospital stay, compared with that of the ESD group. As shown in Table 2, the occurrence rate of GWD in P-STER and ESD group were 58.33% (7/12) and 41.67% (5/7), respectively. In the P-STER group, the mucosa above the surgical wound was retained during and post the procedure, which could protect the wound and abdominal cavity from the irritation of gastric juice. In contract, in the ESD group, the gastric juice and gas could enter the abdominal cavity more easily during the procedure if the GWD occurred, and the postoperative wound would be irritated by the gastric acid if no metal clips were used to close the wound. Therefore, compared with the P-STER group, the patients in the ESD group suffered a higher rate of postoperative abdominal pain, leading to a longer postoperative hospital stay.

Of note, the four patients without complete resection did not receive additional treatments given the postoperative pathologic diagnosis (two were ectopic pancreas and two were G1 GISTs).

The main limitation of this study was that it was designed as a retrospective study. Second, the sample size of this study was small (*n* = 12 for each group) since P-STER was a novel application for prepyloric SMTs in recently. Third, there may be some bias in the choice of treatment strategy according to the operation habits of endoscopists in this study. Moreover, this study relied on reports from a tertiary referral center, and could mean that the results from this study may not be representative of findings from other hospitals. However, to the best of our knowledge, this study first reported the comparison results between P-STER and ESD for prepyloric SMTs.

In conclusion, compared with ESD, we found that P-STER appears to produce a higher operation efficacy. However, further randomized controlled trials were warranted.

## Figures and Tables

**Table 1:** Baseline characteristics of the 24 patients undergoing P-STER or ESD for prepyloric SMTs.

	P-STER (*n* = 12)	ESD (*n* = 12)	*P*-value
Age, mean (range), years	51.00 (28.00–77.00)	46.50 (28.00–60.00)	0.371
Gender (male/female)	5/7	3/9	0.098
Lesion size, median (range), cm	1.75 (1.00–3.00)	1.50 (1.20–3.50)	0.890
Pathology results, *n* (%)			
Ectopic pancreas	5 (41.67)	5 (41.67)	1.000
Gastrointestinal stromal tumor	3 (25.00)	3 (25.00)	
Leiomyoma	1 (8.33)	1 (8.33)	
Lipomyoma	1 (8.33)	1 (8.33)	
Glomangioma	1 (8.33)	1 (8.33)	
Inflammatory mass	1 (8.33)	1 (8.33)	
Distance from pylorus ring, median (range), cm	2.5 (0–3)	2.5 (0–3)	1.000
Location, *n* (%)			
3 o'clock	2 (16.67)	2 (16.67)	1.000
4 o'clock	3 (25.00)	3 (25.00)	
6 o'clock	5 (41.67)	5 (41.67)	
7 o'clock	1 (8.33)	1 (8.33)	
8 o'clock	1 (8.33)	1 (8.33)	
Lesion origin, *n* (%)			
Submucosal layer	5 (41.67)	5 (41.67)	1.000
Muscularis propria layer	7 (58.33)	7 (58.33)	
The appearance under EUS, *n* (%)			
High-medium mixed echo	7 (58.33)	6 (50.00)	0.682
Homogeneous low echo	5 (41.67)	6 (50.00)	

P-STER: prepyloric submucosal tunneling endoscopic resection; ESD: endoscopic submucosal dissection; SMTs: submucosal tumors; EUS: endoscopic ultrasonography.

**Table 2:** Treatment outcomes of P-STER or ESD for prepyloric SMTs.

	P-STER (*n* = 12)	ESD (*n* = 12)	*P*-value
Using the snare during procedure, *n* (%)			
Yes	5 (41.67)	3 (25.00)	0.386
No	7 (58.33)	9 (75.00)	
Closure of the entry or wound using metal clips, *n* (%)			
Yes	12	5 (41.67)	0.002
No	0	7 (58.33)	
En bloc resection, *n* (%)	12 (100.00)	12 (100.00)	1.000
Complete resection, *n* (%)			
Yes	11 (91.67)	9 (75.00)	0.273
No	1 (8.33)	3 (25.00)	
Gastric-wall defect (GWD), *n* (%)			
Yes	7 (58.33)	5 (41.67)	0.414
No	5 (41.67)	7 (58.33)	
Resection time, mean (range), minutes	38.67 (21–57)	52.50 (40–73)	0.001
Recurrence			
Yes	0 (0.00)	0 (0.00)	1.000
No	12 (100.00)	12 (100.00)	
Postoperative bleeding, *n* (%)			
Yes	0 (0.00)	1 (8.33)	0.307
No	12 (100.00)	11 (91.67)	
Postoperative abdominal pain, *n* (%)			
Yes	1 (8.33)	6 (50.00)	0.025
No	11 (91.67)	6 (50.00)	
Postoperative hospital stay, median (range), day	5.50 (5–8)	7.00 (5–9)	0.008
Follow up time, mean (range), months	41.67 (6.00–62.00)	48.25 (20.00–75.00)	0.065
Approximate amount of ESD and STER conducted by operators	≥300	≥300	

P-STER: prepyloric submucosal tunneling endoscopic resection; ESD: endoscopic submucosal dissection; SMTs: submucosal tumors.

## Data Availability

Data supporting this research article are available from the corresponding author or first author on reasonable request.

## References

[1] An W., Sun P. B., Gao J. (2017). Endoscopic submucosal dissection for gastric gastrointestinal stromal tumors: a retrospective cohort study. *Surgical Endoscopy*.

[2] Białek A., Wiechowska-Kozłowska A., Pertkiewicz J. (2012). Endoscopic submucosal dissection for treatment of gastric subepithelial tumors (with video). *Gastrointestinal Endoscopy*.

[3] Zhang H., Huang X., Qu C., Bian C., Xue H. (2019). Comparison between laparoscopic and endoscopic resections for gastric submucosal tumors. *Saudi Journal of Gastroenterology*.

[4] Chai N., Zhang W., Linghu E. Q. (2018). Prepyloric submucosal tunneling endoscopic resection for a case of inflammatory mass (with video). *Digestive Endoscopy*.

[5] Chai N. L., Li H. K., Linghu E. Q. (2019). Consensus on the digestive endoscopic tunnel technique. *World Journal of Gastroenterology*.

[6] Polkowski M. (2005). Endoscopic ultrasound and endoscopic ultrasound-guided fine-needle biopsy for the diagnosis of malignant submucosal tumors. *Endoscopy*.

[7] Hedenbro J. L., Ekelund M., Wetterberg P. (1991). Endoscopic diagnosis of submucosal gastric lesions. The results after routine endoscopy. *Surgical Endoscopy*.

[8] Chung I. K., Lee J. H., Lee S. H. (2009). Therapeutic outcomes in 1000 cases of endoscopic submucosal dissection for early gastric neoplasms: Korean ESD Study Group multicenter study. *Gastrointestinal Endoscopy*.

[9] He G., Wang J., Chen B. (2016). Feasibility of endoscopic submucosal dissection for upper gastrointestinal submucosal tumors treatment and value of endoscopic ultrasonography in pre-operation assess and post-operation follow-up: a prospective study of 224 cases in a single medical center. *Surgical Endoscopy*.

[10] Qi Z. P., Shi Q., Liu J. Z. (2018). Efficacy and safety of endoscopic submucosal dissection for submucosal tumors of the colon and rectum. *Gastrointestinal Endoscopy*.

